# Determining overweight and underweight with a new weight‐for‐height index in captive group‐housed macaques

**DOI:** 10.1002/ajp.22996

**Published:** 2019-06-13

**Authors:** Elisabeth H. M. Sterck, Dian G. M. Zijlmans, Han de Vries, Lisette M. van den Berg, Carel P. van Schaik, Jan A. M. Langermans

**Affiliations:** ^1^ Animal Ecology, Department of Biology Utrecht University Utrecht The Netherlands; ^2^ Animal Science Department Biomedical Primate Research Centre Rijswijk The Netherlands; ^3^ Department of Anthropology University of Zurich Zurich Switzerland; ^4^ Department of Animals in Science and Society, Faculty of Veterinary Medicine Utrecht University Utrecht The Netherlands

**Keywords:** adiposity, body mass index, BMI, colony management, *Macaca*

## Abstract

Housing primates in naturalistic groups provides social benefits relative to solitary housing. However, food intake may vary across individuals, possibly resulting in overweight and underweight individuals. Information on relative adiposity (the amount of fat tissue relative to body weight) is needed to monitor overweight and underweight of group‐housed individuals. However, the upper and lower relative adiposity boundaries are currently only known for macaques living solitarily in small cages. We determined the best measure of relative adiposity and explored the boundaries of overweight and underweight to investigate their incidence in group‐housed adult male and female rhesus macaques and long‐tailed macaques living in spacious enclosures at the Biomedical Primate Research Centre (BPRC), the Netherlands. During yearly health checks different relative adiposity measures were obtained. For long‐tailed macaques, comparable data on founder and wild animals were also available. Weight‐for‐height indices (WHI) with height to the power of 3.0 (WHI3.0) for rhesus macaques and 2.7 (WHI2.7) for long‐tailed macaques were optimally independent of height and were highly correlated with other relative adiposity measures. The boundary for overweight was similar in group‐housed and solitary‐housed macaques. A lower boundary for underweight, based on 2% body fat similar to wild primates, gave a better estimate for underweight in group‐housed macaques. We propose that for captive group‐housed rhesus macaques relative adiposity should range between 42 and 67 (WHI3.0) and for long‐tailed macaques between 39 and 62 (WHI2.7). The majority of group‐housed macaques in this facility have a normal relative adiposity, a considerable proportion (17–23%) is overweight, and a few (0–3%) are underweight.

## HIGHLIGHTS

- Weight‐for‐height indices WHI3.0 for group‐housed rhesus macaques and WHI2.7 for long‐tailed macaques were independent of height.
- The best boundary for underweight in group‐housed macaques was based on 2% body fat of wild primates, rather than 8% body fat of solitary‐housed macaques.

## 1 INTRODUCTION

Group housing of captive primates has beneficial social effects, but also changes other aspects of their life and environment. Group enclosures are more spacious compared with solitary housing situations and individuals have the ability to walk more and thus cover larger distances. Consequently, animals can become more muscular and thus relatively heavy. Moreover, food is typically provided for the whole group and some individuals (especially dominants) may seize the opportunity to take more food than others. All these changes can affect relative adiposity, that is the amount of fat tissue relative to body weight (cf. Benn, 1971). A healthy relative adiposity is not signified by a single value but comprises a range within which animals (or humans) are considered to have a healthy weight (Raman et al., 2005). Individuals above the upper boundary are considered overweight, whereas those below the lower boundary are considered underweight. Both being overweight or underweight have empirically documented adverse effects on the health and welfare of an individual (Kemnitz & Francken, 1986; Scarlett & Donoghue, 1998; Shively & Clarkson, 1987). However, there is no agreed‐upon measure of relative adiposity and boundaries of over‐ and underweight for group‐housed macaques. Current measures of relative adiposity and subsequent boundaries are based on solitary‐housed macaques living in small cages (e.g., Raman et al., 2005) and these may be different for group‐housed macaques.

The first challenge is how to measure relative adiposity. A valid measure fulfills two criteria: (a) its distribution should be independent of height; and (b) it should be highly correlated with other measures of relative adiposity (Benn, 1971). Relative adiposity is often measured with weight‐for‐height indices (WHI) that scale body weight to a power of height (Benn, 1971). A specific WHI measure, the body mass index (BMI), is well‐known in humans. BMI is calculated by dividing body weight by the square of the height and can be coded WHI2.0 (indicating that the power of height is 2.0; Keys, Fidanza, Karvonen, Kimura, & Taylor, 1972). BMI is generally independent of height in adult humans, but there is discussion whether the power of height is always two (Heymsfield, Gallagher, Mayer, Beetsch, & Pietrobelli, 2007). The Ponderal index is a WHI measure where body weight is normalized with the third power of height (weight/height^3.0^; Rohrer, 1921). As using an inappropriate WHI can produce misleading results about the relative adiposity, some authors state that the power of height is population specific; this is incorporated in the Benn index (weight/height^β^; Benn, 1971; Lee, Kolonel, & Hinds, 1982). The β can be population specific when populations differ in body build and ideally should be calculated for each population separately.

Additional measures of relative adiposity, besides WHI, concern body circumferences and skinfold thicknesses (Bodkin, Hannah, Ortmeyer, & Hansen, 1993; Colman, Hudson, Barden, & Kemnitz, 1999; Hamada, Hayakawa, Suzuki, Watanabe, & Ohkura, 2003; Kemnitz & Francken, 1986; Kemnitz, Goy, Flitsch, Lohmiller, & Robinson, 1989; Walker, Schwartz, Wilson, & Musey, 1984). Relative adiposity has also been measured by body condition scoring (BCS). BCS uses palpation of key anatomic features such as hips, spine, pelvis, thorax, and abdomen and can be easily incorporated into routine health checks (Clingerman & Summers, 2005). This measure is used in a wide variety of animal species, including horses, cats, dogs, sheep, mice, and cattle (Carroll & Huntington, 1988; German, Holden, Moxham, & Holmes, 2006; Thompson & Meyer, 1994; Ullman‐Culleré & Foltz, 1999; Wildman et al., 1982). BCS has been validated in rhesus macaques (Summers, Clingerman, & Yang, 2012).

The second challenge is to determine the boundaries of over‐ and underweight for group‐housed macaques. In humans, the relationship between body fat percentage and BMI is background‐specific due to variation in trunk‐to‐leg length, slenderness, and/or muscularity (Deurenberg, Deurenberg‐Yap, & Guricci, 2002). As a consequence, different BMI boundaries have to be considered (Deurenberg et al., 2002) and this may also apply to different primate populations. Raman et al. (2005) determined BMI boundaries for male and female rhesus macaques based on fat reserves in relatively old and solitary‐housed animals living in small cages. The upper boundary was based on the insulin sensitivity index, which yielded an upper boundary of 23% body fat for males and 18% for females. The lower boundary was based on the body fat percentage below which individual health could quickly deteriorate, which yielded 9% body fat for males and 8% for females but included a large safety margin. Alternatively, the lower boundary can be based on the fat percentage of wild primates, for example, 1.9% in baboons and 2.1% in toque macaques (Altmann, Schoeller, Altmann, Muruthi, & Sapolsky, 1993; Dittus, 2013). The latter boundary (ca 2%) may be more appropriate for group‐housed macaques living in relatively large enclosures, as they are more similar to wild than solitary‐housed animals. In addition, boundaries can be based on deviation from the mean (Schwartz, Kemnitz, & Howard, 1993). Finally, the BCS also has boundary values for overweight (>3.5) and underweight (<2.5; Clingerman & Summers, 2005; Summers et al., 2012).

The goal of the present study was to determine the best measure of relative adiposity and explore the boundaries of overweight and underweight to investigate their incidence in captive group‐housed adult rhesus macaques (*Macaca mulatta*) and long‐tailed macaques (*M. fascicularis*) living in spacious enclosures. For the long‐tailed macaques comparable data on founder and wild animals were available. We determined species‐specific WHI measures independent of height and correlated these with other measures of relative adiposity. We also used several methods to determine the upper and lower boundaries of relative adiposity and derived the appropriate boundaries for group‐housed macaques.

## 2 MATERIALS AND METHODS

### 2.1 Subjects and housing current population BPRC

The subjects of this study were 300 adult rhesus macaques and 105 adult long‐tailed macaques housed in social groups at the BPRC in Rijswijk, the Netherlands. Females older than 6 years of age and males older than 8 years of age were defined as adults, as they are skeletally mature (Schwartz et al., 1993; see Figure S1). Only nonpregnant females were included in the data set, because pregnant females are known to have higher body weight, BMI and abdominal circumferences than nonpregnant females in free‐ranging rhesus macaques (Schwartz & Kemnitz, 1992). Pregnancy was detected during the yearly health check or was determined afterwards as the 6 months preceding an infant's birth.

Four female rhesus macaques older than 25 years of age were excluded from the analyses, because elderly macaques may experience aging, just like humans, in which changes in physiology and metabolism are accompanied by changes in body size and weight (Ramsey, Laatsch, & Kemnitz, 2000; Table S1). In the end, 273 female and 23 male rhesus macaques were included in the analyses, whereas the long‐tailed macaque sample contained 92 females and 13 males housed at the BPRC. All monkeys were captive‐born.

Social groups at the BPRC typically consist of 15–40 individuals and encompass several matrilines, that is females with descendants, and one non‐natal adult male. Husbandry is aimed at mimicking natural demographic processes. Each social group had access to enriched (Vernes & Louwerse, 2010) indoor (±108 m^2^ and 2.85 m high) and outdoor (±260 m^2^ and 3.1 m high) enclosures. The monkeys were fed monkey chow (Sniff^©^) on a daily basis, complemented with fruit, vegetables, or bread. The amount of food was predetermined per group and linked to the summed needs of all individuals. Water was available ad libitum.

### 2.2 Subjects and housing other long‐tailed macaque populations

In addition to the BPRC population, 24 founder female and 11 founder male long‐tailed macaques were subjected to anthropometric measurements at the Utrecht University from 1987 to 1989. These founders were part of the population from which the current BPRC long‐tailed macaques descended. Housing and feeding were comparable with the current BPRC conditions.

Data from the wild were available for nine female and six male long‐tailed macaques from the Ketambe Research Station, Gunung Leuser National Park, Indonesia in 1989. The wild long‐tailed macaques concerned individuals from the H‐group and were all healthy (van Noordwijk & van Schaik, 1999).

### 2.3 Anthropometric measurements current BPRC population

Relative adiposity levels of BPRC individuals were estimated by taking several anthropometric measures (Table S2; cf. Garcia, Huffman, & Shimizu, 2010) when the animals were sedated during their yearly health check. The yearly health checks are a veterinary management procedure at the BPRC colony. No invasive research or experimental procedures requiring ethics approval according to the European Directive 2010/63 and the Dutch law were performed. Therefore, no approval by the BPRC animal ethics committee was required. This study is consistent with the ASP Principles for the Ethical Treatment of Non‐human Primates.

The measurements took place between 9.00 and 14.00 hr from November 4, 2014 until May 10, 2017. Anthropometric measures concerned body weight, height, abdominal circumference, and skinfold thickness at four sites. All measurements were performed on the animal's right side of the body. The measurements were performed in three subsequent years. Within any given year, one person was responsible for performing all the measurements. Whenever an individual was measured in more than 1 year, the average value was used for the analyses.

Body weight was measured with a standard scale and was expressed in kilograms accurate to one decimal. Height was measured as the crown‐rump length, that is the distance from the highest point on the head to the bottom of the monkey. Height was measured using a SECA 210 measuring mat for human infants (Seca, Hamburg, Germany). The monkey was in a supine position and height was determined to the nearest 0.1 cm. Weight and height were used to calculate BMI and WHI measures. Abdominal circumference was measured at the height of the umbilicus with a tape measure to the nearest 0.1 cm with the animal in the lateral recumbent position (Colman et al., 1999). Skinfold thickness was measured three times to the nearest 0.1 mm with a Baseline Pro skinfold caliper at four different sites, namely abdominal (at the height of the umbilicus), subscapular (1 cm below the inferior angle of the scapula), supra‐iliac and at the triceps. A total skinfold thickness score was calculated by summing the average skinfold thicknesses at the four sites.

Furthermore, all macaques were subjected to BCS (Summers et al., 2012), which was performed by experienced veterinarians. In rhesus macaques, the BCS scale ranges from 1.0 to 5.0 comprising both whole and half units, in which the midrange (3.0) represents optimal body condition. Lower values (<2.5) represent emaciated to lean conditions and higher values (>3.5) indicate excessive body fat (Clingerman & Summers, 2005). This BCS system was also applied to the long‐tailed macaques.

### 2.4 Anthropometric measurements on founder and wild long‐tailed macaques

Anthropometric measurements on the founder and wild long‐tailed macaques were performed by CPvS (Table S2). Measurements on the founder long‐tailed macaques were performed every few months between December 1987 and April 1989, with some slight deviations from the measurements in 2014–2017. Data from six body weight measurements and five height measurements were averaged to yield mean values for every individual. Data were not corrected for pregnancies. Similar to the current data, the body weight was measured with a standard scale and was expressed in kilograms accurate to one decimal. Differently from the current data, the height was measured by seating the animal and measuring the distance from its bottom to its head. This measure is similar to our crown‐rump length. The abdominal circumference and skinfold thickness at four sites were based on two data points and measured similar to the current data. Skinfold thickness was calculated from the average of two measures.

Anthropometric measurements performed on the wild long‐tailed macaques were the same as for the founder population. These measurements were performed once (February 1989).

### 2.5 Defining overweight and underweight

Five different methods were used to determine whether individuals were overweight or underweight: (a) BMI (=WHI2.0); (b) WHI; (c) abdominal circumference; (d) standard deviation (SD) from the mean WHI; and (e) BCS. WHI and SD from the mean WHI were calculated for this study, whereas the other boundaries were based on literature. First, the upper and lower BMI boundaries in solitary‐housed rhesus macaques are between 32 and 44 kg/m^2^ for males and between 27 and 35 kg/m^2^ for females (Raman et al., 2005). We also applied these boundaries to the long‐tailed macaques, as for females and males, respectively, the BMI did not differ significantly between the species (females: Mann–Whitney *U* test, *U* = 107,45.5, *n* = 362, *p* = .102; males: Mann–Whitney *U* test, *U* = 118, *n* = 36, *p* = .312). Second, the upper and lower boundaries of the WHI3.0 and WHI2.7 we obtained (see Section 3) were calculated on the basis of BMI boundaries (Raman et al., 2005; see Supporting Information for data conversion). Likewise, we used the 2% body fat similar to wild primates (Altmann et al., 1993; Dittus, 2013) for a female of average height to determine the lower boundary. Third, we used Raman et al.'s (2005) lower and upper boundaries for solitary‐housed rhesus macaques for abdominal circumference: 40 and 54 cm for adult males and 35 and 44 cm for adult females, respectively. These were also applied to long‐tailed macaques. Fourth, overweight and underweight were determined as being more than two standard deviations above and below the mean WHI (Schwartz et al., 1993). The fifth method used was BCS (Summers et al., 2012), which defines underweight as BCS < 2.5 and overweight as BCS > 3.5 (Summers et al., 2012).

### 2.6 Statistical analyses

The WHI measure that was least correlated with height was determined by calculating the appropriate power β in the formula WHI = weight/height^β^ as determined using a custom program in R Core Team (2015). This program searches for the β that yields the regression coefficient closest to 0 for the regression line of WHI regressed on height. The value of β was determined separately for female rhesus macaques and female long‐tailed macaques. The 95% confidence limits of each β were determined by means of the nonparametric bias‐corrected accelerated (BCa) method using the function “bcanon” from the package “bootstrap” in R version 3.2.3 (2015; Efron & Tibshirani, 1993).

The different anthropometric measurements of each species, sex and population were correlated using Spearman's correlations. Sex differences in rhesus macaques were tested using independent samples *t* tests or Mann–Whitney *U* tests, depending on whether the data were normally distributed. In the long‐tailed macaques, separate analyses were conducted for population and sex differences, because the differences in variance did not allow a combined (i.e., two‐way ANOVA) analysis. Differences between the populations were determined with a one‐way ANOVA or Kruskal–Wallis *H* test, depending on whether the data were normally distributed. Sex differences were tested in the same way as in the rhesus macaques. Normal distribution of the data was tested using the Shapiro–Wilk test. Statistical analyses were performed in IBM SPSS Statistics version 22 and the significance level used in this study was *α* = .05. All statistical tests were two‐tailed.

## 3 RESULTS

### 3.1 Determining macaque WHI

**Figure 1:**
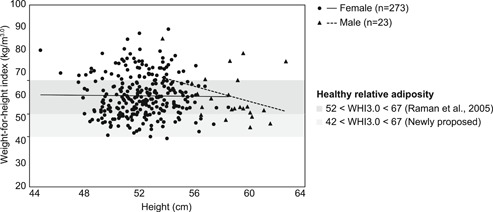
WHI3.0 plotted against height for adult female (black circles) and male (black triangles) rhesus macaques currently housed at the BPRC. WHI3.0 was optimally independent of height in females (black solid line), whereas there was a nonsignificant negative relationship between WHI3.0 and height in males (black dashed line). The dark gray bar represents the proposed relative adiposity boundaries by Raman et al. (2005), which correspond to 52 < WHI3.0 < 67. The light gray bar indicates the new lower boundary based on 2% body fat, similar to wild primates. BPRC, Biomedical Primate Research Centre; WHI, weight‐for‐height indices

**Figure 2:**
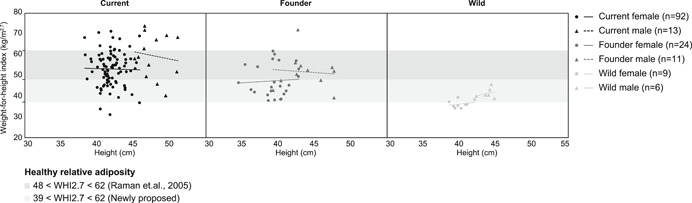
WHI2.7 plotted against height for different adult long‐tailed macaque samples. First panel: Current BPRC females (black circles) and current BPRC males (black triangles); second panel: founder females (dark gray circles) and founder males (dark gray triangles); and third panel: wild females (light gray circles) and wild males (light gray triangles). The dark gray bar represents the proposed relative adiposity boundaries by Raman et al. (2005), which correspond to 48 < WHI2.7 < 62. The light gray bar indicates the new lower boundary based on 2% body fat, similar to wild primates. WHI2.7 values of different sex‐population groups were generally independent of height. WHI, weight‐for‐height indices

The ideal WHI should be independent of height. WHI was calculated for females of each macaque species, as most data points were available on adult females of the current BPRC population and the sample size of adult males was small. BMI was significantly correlated with height in female rhesus macaques (Spearman's correlation, *r* = .276, *n* = 273, *p* < .0005; Figure S2). Although only a trend, a positive slope was also found for female long‐tailed macaques (Pearson's correlation, *r* = .201, *n* = 89, *p* = .059; Figure S3). The WHI that was least correlated with height was determined. The correlation for rhesus macaque females was found to be closest to zero at a WHI with height to the power of 2.96 (rounded to 3.0; 95% confidence interval [CI] = [2.53, 3.39]) and for long‐tailed macaque females 2.73 (rounded to 2.7; 95% CI = [1.76, 3.55]; Figures 1, 2: black circles and black regression lines).

**Figure 3:**
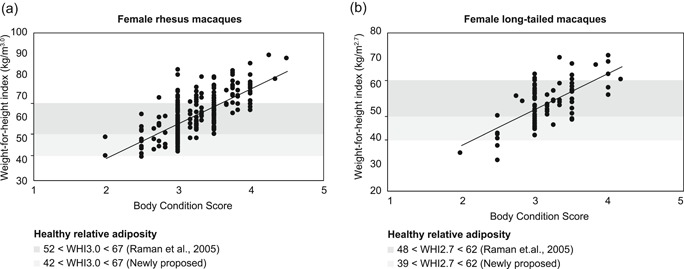
Relationship between body condition score and (a) WHI3.0 (rhesus macaque females) and (b) WHI2.7 (long‐tailed macaque females) at the BPRC. The dark gray bar represents the proposed relative adiposity boundaries by Raman et al. (2005), which are 52 < WHI3.0 < 67 in rhesus macaques and 48 < WHI2.7 < 62 in long‐tailed macaques. The light gray bar indicates the new lower boundary based on 2% body fat levels in the wild, which corresponds to WHI3.0 = 42 (rhesus macaques) and WHI2.7 = 39 (long‐tailed macaques). The *y*‐axes are differently scaled. BPRC, Biomedical Primate Research Centre; WHI, weight‐for‐height indices

The other relative adiposity measures, that is abdominal circumference, skinfold thickness, and BCS, were all highly correlated with BMI, WHI3.0 (rhesus macaques) and WHI2.7 (long‐tailed macaques; (Figure 3a,b; Tables S3 and S4). Given that the WHI3.0 and WHI2.7 were independent of height (which BMI is not) and were highly correlated with other relative adiposity measures, we propose that these are better estimates of relative adiposity than the BMI (WHI2.0) for these macaques.

### 3.2 Relative adiposity in males and other populations

The general applicability of WHI3.0 (rhesus) and WHI2.7 (long‐tailed macaques) was tested per species to see whether these measures also apply to adult males (both species) and to different population samples (long‐tailed macaques).

#### 3.2.1 Sex differences in rhesus macaques

In male rhesus macaques, there was an almost significant negative relationship between height and WHI3.0 (Spearman's correlation, *r* = −.380, *n* = 23, *p* = .074; Figure 1), whereas WHI3.0 was highly correlated with other relative adiposity measures (Table S3). Next, we compared female and male characteristics (Table S7, statistics). Male rhesus macaques had a significantly higher body weight and height than females. Abdominal circumference was significantly higher in males, while females had higher subscapular skinfold thickness. Abdominal skinfold thickness, supra‐iliac skinfold thickness, triceps skinfold thickness, total skinfold thickness and BCS did not differ between the sexes. Male rhesus macaques had significantly higher BMI compared with females, whereas the sexes did not differ in WHI3.0.

#### 3.2.2 Correlations of adiposity measures in males and different long‐tailed macaque populations

In the current male long‐tailed macaque population, WHI2.7 did not depend significantly on height (Pearson's correlation, *r* = −.132, *n* = 13, *p* = .668; Figure 2) and was highly correlated with other relative adiposity measures (Table S4). Similarly, WHI2.7 did not depend significantly on height (Figure 2) in founder females (Spearman's correlation, *r* = .051, *n* = 24, *p* = .813), founder males (Pearson's correlation, *r* = −.090, *n* = 11, *p* = .793), wild females (Pearson's correlation, *r* = .231, *n* = 9, *p* = .550) and wild males (Pearson's correlation, *r* = .273, *n* = 6, *p* = .600). Moreover, WHI2.7 was highly correlated with other relative adiposity measures in the founder population (Table S5). In the wild population, only a few relative adiposity measures were significantly correlated with WHI2.7 (Table S6).

#### 3.2.3 Population and sex differences in long‐tailed macaques

For each sex, we examined variation in various body measures among the current, founder and wild populations (Figure 2; Table S7, statistics). In both sexes, body weight, height, abdominal circumference, subscapular skinfold thickness, supra‐iliac skinfold thickness, triceps skinfold thickness, and total skinfold thickness were higher for the current population compared with the founder and wild population. Furthermore, female BMI and WHI2.7 were higher in the founder population compared with the wild population. Abdominal skinfold thickness, BMI and WHI2.7 of males were significantly higher in the current than wild population, but did not differ from the founder population.

Sex differences were tested for the different populations separately (Table S7, statistics). The sexes did not differ significantly in abdominal circumference in all three populations. Males had higher body weight, height, and BMI than females in all three populations. Males had significantly higher WHI2.7 compared with females in the current and wild population, but not the founder population. Skinfold thicknesses were higher for males from the wild population than females. Similarly, current BPRC male triceps thickness and the founder male total skinfold thickness was significantly higher than for females, yet other male and female skinfold thicknesses did not differ in the current and founder populations. BCS did not differ significantly between the sexes in the current BPRC population.

### 3.3 Estimating boundaries of overweight and underweight with the new WHI measures

The new relative adiposity measures, WHI3.0 for rhesus macaques and WHI2.7 for long‐tailed macaques, can be applied to both females and males and, for long‐tailed macaques, to different populations. We, therefore, calculated boundaries of overweight and underweight on the basis of these measures. This resulted in WHI boundaries (cf. Raman et al., 2005 for solitary‐housed macaques), with a lower boundary based on 8–9% body fat, for rhesus macaques of 52 < WHI3.0 < 67 and for long‐tailed macaques 48 < WHI2.7 < 62. The lower WHI boundary based on 2% body fat, similar to wild primates, was 42 for rhesus macaques (WHI3.0) and was 39 for long‐tailed macaques (WHI2.7).

### 3.4 Estimating the incidence of overweight and underweight

The incidence of overweight and underweight in the current BPRC population was determined employing five different methods (Table 1). Depending on the method, estimates of overweight percentages in the population varied between 4.1% and 31.8% in rhesus macaques and between 2.0% and 24.8% in long‐tailed macaques. Methods 1, 2, and 5 provided intermediate estimates of the percentages of overweight in both species. Method 3 provided a high proportion, whereas Method 4 provided a low proportion of overweight.

**Table 1:** Incidence of overweight and underweight in the BPRC breeding colony per species‐sex group assessed with different methods

	Incidence of overweight					Incidence of underweight					
	M1: BMI boundaries	M2: WHI boundaries ^a^	M3: AC boundaries	M4: mean WHI ±2 *SD*	M5: BCS	M1: BMI boundaries	M2: WHI boundaries with 8% body fat ^a^ ^,^ ^b^	M2: WHI boundary with 2% body fat^c^	M3: AC boundaries	M4: mean WHI ±2 *SD*	M5: BCS
Criterion	♂: BMI > 42	RM: WHI3.0 > 67	♂: AC > 54	♂♀: WHI > mean + 2 *SD*	♂♀: BCS > 3.5	♂: BMI < 32	RM: WHI3.0 < 52	RM: WHI3.0 < 42	♂: AC < 40	♂♀: WHI < mean −2 *SD*	♂♀: BCS < 2.5
	♀: BMI > 35	LTM: WHI2.7 > 62	♀: AC > 44			♀: BMI < 27	LTM: WHI2.7 < 48	LTM: WHI2.7 < 39	♀: AC < 35		
*Rhesus macaques*											
Males (♂)	4/23	6/23	4/23	1/23	5/23	8/23	5/23	0/23	6/23	0/23	2/23
	17.4%	26.1%	17.4%	4.3%	21.7%	34.7%	21.7%	0%	26.1%	0%	8.7%
Females (♀)	60/273	62/273	89/269	11/273	40/273	59/273	56/273	1/273	53/269	1/273	2/273
	22.0%	22.7%	33.1%	4.0%	14.7%	21.6%	20.5%	0.4%	19.7%	0.4%	0.7%
Total	64/296	68/296	93/292	12/296	45/296	67/296	61/296	1/296	59/292	1/296	4/296
	21.6%	23.0%	31.8%	4.1%	15.2%	22.6%	20.6%	0.3%	20.2%	0.3%	1.4%
*Long‐tailed macaques*											
Males (♂)	4/13	6/13	1/12	0/13	3/13	3/13	2/13	0/13	5/12	0/13	0/13
	30.8%	46.2%	8.3%	0%	23.1%	23.1%	15.4%	0%	41.7%	0%	0%
Females (♀)	13/88	11/ 88	24/89	2/88	6/92	24/88	23/88	3/88	12/89	3/88	1/92
	14.8%	12.5%	27.0%	2.2%	6.5%	27.3%	26.1%	3.4%	13.5%	3.4%	1.1%
Total	17/101	17/101	25/101	2/101	9/105	27/101	25/101	3/101	17/101	3/101	1/105
	16.8%	16.8%	24.8%	2.0%	8.6%	26.7%	24.8%	3.0%	16.8%	3.0%	1.0%

*Notes*: The boundaries were based on the literature. Method 1: BMI boundaries (Raman et al., 2005); Method 2: WHI boundaries per species; Method 3: abdominal circumference boundaries (Raman et al., 2005); Method 4: based on two standard deviations from the mean WHI per species (Schwartz et al., 1993); Method 5: based on BCS (Clingerman & Summers, 2005).

Abbreviations: AC, abdominal circumference; BCS, body condition scoring; BMI, body mass index; BPRC, Biomedical Primate Research Centre; *SD*, standard deviation; WHI, weight‐for‐height indices.

^a^ The WHI measures of rhesus macaque males and females did not differ significantly (Table S7). The WHI measures of long‐tailed macaque males were higher than of females (Table S7), but we could not determine a boundary. Therefore, we calculated the WHI per species and used the same (female) boundary for both sexes.

^b^ Based on Raman et al. (2005).

^c^ Based on body fat of wild primates (Altmann et al., 1993; Dittus, 2013).

The incidence of underweight varied between 0.3% and 22.6% in rhesus macaques and between 1.0% and 26.7% in long‐tailed macaques. Methods 1, 2 (based on 8% body fat) and 3 resulted in a large proportion of underweight individuals. Methods 2 (based on 2% body fat), 4 and 5 provided relatively low proportions of underweight.

## 4 DISCUSSION

We determined the best measure of relative adiposity and explored the boundaries of overweight and underweight in captive group‐housed rhesus and long‐tailed macaques. The WHI with height to the power of 3.0 (rhesus macaques) and 2.7 (long‐tailed macaques) depended least on height and were highly correlated with other relative adiposity measures. Therefore, we considered these WHI measures better than the BMI (i.e., WHI2.0). The percentages of overweight and underweight individuals were estimated with five different methods, based on upper and lower boundaries derived from the literature. These showed large differences in their outcomes. The upper WHI boundary based on solitary‐housed macaques (cf. Raman et al., 2005) gave an intermediate incidence of overweight and may apply to group‐housed macaques. In contrast, the lower boundary proposed for solitary‐housed macaques resulted in a large percentage of underweight individuals. A lower boundary based on 2% body fat of wild primates yielded few underweight individuals and may constitute a better estimate of the incidence of underweight in group‐housed macaques.

### 4.1 The best measure of WHI in captive group‐housed macaques

A WHI can be used to measure relative adiposity, but the power of height may be population specific (Benn, 1971). In the BPRC population BMI was positively correlated with height in both female rhesus and female long‐tailed macaques. Therefore, BMI is not the best measure of relative adiposity in these macaques. The WHI that correlated least with height was determined. This differed between the two macaque species: it was WHI3.0 (or the Ponderal index, weight/height^3^) for rhesus macaques and WHI2.7 (weight/height^2.7^) for long‐tailed macaques. Both WHIs were highly correlated with other adiposity measures, that is abdominal circumference, skinfold thicknesses and BCS. Altogether, WHI3.0 for rhesus and WHI2.7 for long‐tailed macaques fit the two criteria for a valid measure of relative adiposity.

In rhesus macaques, sex differences in BMI indicated that males had a significantly higher relative adiposity than females, whereas no such difference was found for WHI3.0. However, males were also taller, and the correlation of BMI with height may have been responsible for this outcome. Indeed, most other adiposity measured did not show a sex difference. Similarly, in the founder long‐tailed macaques WHI2.7 did not show a sex difference, whereas BMI did. This indicates that using a WHI that depends on height can result in spurious outcomes. However, WHI2.7 and BMI of the current BPRC and wild long‐tailed macaques indicated that males had a higher relative adiposity than females. This suggests a population‐specific sex difference in WHI2.7 in long‐tailed macaques.

As for both macaque species the “traditional” BMI measure was not the best way to measure relative adiposity, this may also apply to other macaque species. The two study species differed in the power of height that gave the best estimate: for rhesus macaques WHI3.0 and for long‐tailed macaques WHI2.7. The difference between the species may be related to the more terrestrial habits of rhesus macaques and the mostly arboreal habits of long‐tailed macaques that have resulted in relatively robust rhesus and slender long‐tailed macaques (Cant, 1988; Rodman, 1979). Alternatively, the differences may be due to body size. However, the two species do overlap in height. Moreover, the CIs include a large range of power estimates, especially in long‐tailed macaques due to the smaller sample size. Ideally, the power of WHI should be determined for each species separately, yet this requires large sample sizes. When this cannot be calculated, we suggest that, depending on the robustness of the species and their terrestrial or arboreal lifestyle, the rhesus or long‐tailed macaque WHI measure should be used.

### 4.2 WHI3.0 in rhesus macaques

Although the WHI3.0 was determined for female rhesus macaques, males also had measures within the female range. Similarly, the WHI3.0 and most other relative adiposity measures did not differ systematically between the sexes, suggesting that these measures did not depend on sex. The suggestion that WHI3.0 is also the best measure for males seems contradicted by the almost significant negative relationship between male height and WHI3.0 (see also Figure 1). However, the number of males was relatively low (*n* = 23) and this outcome hinged on one exceptionally short and stocky individual. When excluding this nonrepresentative male, a weak relationship between male height and WHI3.0 was found (Spearman correlation's, *r* = −.296, *n* = 22, *p* = .182). Currently, WHI3.0 seems a good measure of relative adiposity in both full‐grown female and male rhesus macaques. Future research should aim to estimate the power of height in WHI for rhesus macaque males based on a larger data set.

### 4.3 WHI2.7 in long‐tailed macaques

For female long‐tailed macaques, the WHI estimates of the current BPRC population could be compared with other samples, namely the founders of the current BPRC population and wild long‐tailed macaques. In all populations and in both sexes, WHI2.7 was independent of height. Moreover, WHI2.7 was correlated with other adiposity measures. Therefore, WHI2.7 seems a measure of relative adiposity applicable to all measured populations of long‐tailed macaques.

The founder and the wild individuals were smaller, lighter and had a lower WHI2.7 than the current BPRC animals, whereas founder and wild individuals were similar in many of the adiposity measures. The animals in the current population being taller than the founder and wild animals suggest that they may obtain maximum length in captive conditions with optimal food and few diseases, or that captive management unintentionally selected for taller animals. We cannot distinguish between these two options. The comparison of the WHI2.7 and other relative adiposity measures of the current BPRC population with the founders and wild macaques suggests that the current BPRC population is relatively heavy.

Male long‐tailed macaques of the current BPRC population were compared with the females. Males were larger, heavier and had a higher WHI2.7 than females. They had a higher triceps skinfold thickness than females as well, but the other relative adiposity measures did not differ between the sexes. This may have several explanations. First, the male long‐tailed macaques were from a different genetic origin than the females, to prevent inbreeding. These populations may have a different relative adiposity. Second, the higher male WHI2.7 may reflect a relatively high muscle mass. As most relative adiposity measures of current BPRC males and females did not differ, this suggests that males may indeed be more muscular. We did not find a similar effect in the other populations. In the founder population, females and males were similar in most relative adiposity measures, whereas in the wild population males had higher adiposity than females. This may either indicate a real difference between the populations or can be due to the small sample sizes.

### 4.4 The incidence of overweight and underweight

We determined the incidence of overweight and underweight in the current BPRC populations based on five different methods. The different measures showed highly variable outcomes.

Method 1 (Raman et al., 2005) determined BMI boundaries for male and female rhesus macaques based on fat reserves in relatively old and solitary‐housed animals living in small cages. The estimates for the percentage of overweight individuals were intermediate between the other measures, yet the estimates for underweight were high. This may have two explanations. First, many of the studied animals may be underweight (see below). Second, the boundary for underweight may be set at a relatively high value. Indeed, Raman et al. (2005) based the lower boundary on 8–9% body fat and included a large safety margin (3%). Therefore, the lower boundary for solitary‐housed rhesus macaques may not represent the correct reference values for group‐housed macaques that have more opportunities to move around in their enclosures.

The calculations for the WHI boundaries in Method 2 were based on the BMI measures of Raman et al. (2005) and give a similar pattern in their estimations of overweight and underweight. Like for Method 1, the lower boundary of WHI based on 8–9% body fat resulted in a large percentage of underweight individuals. Alternatively, when considering a lower boundary based on 2% body fat in wild primates (Altmann et al., 1993; Dittus, 2013), the percentage of underweight individuals becomes similar to the (low) estimates of two other measures (rhesus macaque females: 0.3%; long‐tailed macaque females: 3.0%). Based on 2% body fat the lower boundary for underweight is WHI3.0 = 42 for rhesus macaques and WHI2.7 = 39 for long‐tailed macaques.

Method 3 was based on the boundaries of abdominal circumference for solitary‐housed male and female rhesus macaques (Raman et al., 2005). This resulted in many overweight and many underweight individuals for both rhesus and long‐tailed macaques. This method was not in line with the overall pattern and probably overestimates problematic weights.

Method 4 (Schwartz et al., 1993) is based on the population average in WHI and its variation. This method gives the lowest proportion of overweight and underweight individuals in both species, although more individuals are overweight than underweight. Some individuals had weights above the normal variation, arguing that overweight does exist in both species. In addition, only three female long‐tailed macaques and one female rhesus macaque have a value below the normal variation, indicating that underweight is rare in this population. However, a weakness of this method is that it depends on the population average: when all individuals are relatively heavy, relatively few individuals will be considered overweight and vice versa. The boundary for underweight yields WHI3.0 = 42 for rhesus monkeys and WHI2.7 = 37 for long‐tailed macaques.

Method 5 measuring the BCS (Clingerman & Summers, 2005) is based on expert evaluation of body fat and muscle tissue and uses palpation of key anatomic features. Similar to Methods 1 and 2, this method resulted in intermediate percentages of overweight, whereas the percentage of underweight individuals was very low. Therefore, this method suggests that overweight is found in these macaques, but that underweight is rare. When the optimal body condition (BCS = 3) was used as a reference to create WHI boundaries, rhesus macaques have an optimal relative adiposity range between 44 < WHI3.0 < 82 and long‐tailed macaques between 41 < WHI2.7 < 64.

### 4.5 Proposed WHI boundaries for overweight and underweight

Based on estimates of the five methods, we propose WHI boundaries for group‐housed macaques that live in relatively large enclosures with inside and outside compartments. For overweight, we propose to follow the intermediate values from Method 2 (based on Method 1) to determine the WHI boundary. This results for rhesus macaques in WHI3.0 = 67 and for long‐tailed macaques in WHI2.7 = 62. This is the same boundary as proposed previously by Raman et al. (2005) for solitary‐housed macaques. They based their upper boundary on health considerations. Whether this also applies to group‐housed macaques remains to be established.

For underweight, we propose to follow Methods 4 and 5 and the boundary of Method 2 when using 2% body fat (Altmann et al., 1993; Dittus, 2013). This leads to very few underweight individuals in the current BPRC population (rhesus macaques: 0.3%; long‐tailed macaques: 3.0%) and fits the observation that females with a low WHI give birth to offspring at a normal rate (non‐published data). In addition, individuals with a low WHI were typically considered “normal” (i.e., BCS = 3) with the BCS method. This also complies with the impression that individuals with a low WHI are similar in build to reproducing wild long‐tailed macaques (EHMS personal observation; cf. Altmann et al., 1993; Dittus, 2013). Actually, most females of the wild population had an even lower WHI2.7. This suggests that the lower boundary is not stricter than living in the wild. Higher boundaries would consider “normally” slender individuals underweight. In addition, a relatively low weight in macaques may improve longevity and not necessarily be unhealthy (Mattison et al., 2017). For rhesus macaques, the estimates of Methods 2 and 4 are similar and propose a WHI3.0 = 42 (Figure 3a). For long‐tailed macaques, Method 2 results in WHI2.7 = 39, whereas Method 4 results in WHI2.7 = 37, we propose to use the more conservative WHI2.7 = 39 as the lower boundary (Figure 3b).

The BCS (Method 5) was the only method that yielded both an intermediate incidence of overweight and the proposed proportion of underweight individuals. The BCS correlated with all measures indicating adiposity as well. However, BCS also correlated positively with height, which is undesirable. In addition, the experts who determined BCS seemed to vary in how they applied the BCS system, some being more conservative than others (unpublished data). As a result, animals with an optimal BCS (BCS = 3) vary greatly in WHI (Figure 3) and some were even considered overweight based on WHI measures. In contrast, some individuals considered overweight based on their BCS (BCS > 3.5), had a normal relative adiposity when based on WHI measures. Therefore, the two methods do not agree. We propose to use the WHI estimates as it is relatively objective and precise, identifying individuals near the higher or lower boundary of the normal WHI range. Moreover, monitoring of individual‐specific relative adiposity between different measuring moments can be more precise.

In conclusion, relative adiposity in macaques is best measured for rhesus macaques with WHI3.0 (weight/height^3^) and for long‐tailed macaques WHI2.7 (weight/height^2.7^), as these WHI measures are independent of height and are highly correlated with other relative adiposity measures. We propose that a healthy relative adiposity in captive group‐housed rhesus macaques ranges between 42 < WHI3.0 < 67 and in long‐tailed macaques between 39 < WHI2.7 < 62. The lower boundary is based on fat percentages similar to wild primates, whereas the upper WHI boundary complies with a previously proposed boundary for overweight in solitary‐housed rhesus macaques (i.e., Raman et al., 2005). The more objective identification of over‐ and underweight via appropriate WHI measures may aid in more focussed clinical and husbandry decisions for macaques. The use of the established upper boundary and this new lower boundary results in an acceptable weight for the majority of the group‐housed macaques in spacious enclosures with very few underweight animals and a considerable proportion of overweight animals. Further research into health parameters in group‐housed individuals with high and low WHI values is still required.

## Supporting information

Supporting information
