# Supplementary material for: Determining overweight and underweight with a new weight‐for‐height index in captive group‐housed macaques
Source: Am J Primatol. 2019 Jun 13;81(6):e22996. doi: 10.1002/ajp.22996 (PMC6772146; doi:10.1002/ajp.22996)
Supplement: Supplementary file 1 — Supporting information [file AJP-81-na-s001.docx]

Supplementary information

*Age – height relationship*

We checked the premise from the literature that females older than six years of age can be defined as skeletally mature in the BPRC population (Schwartz et al., 1993). Surprisingly, adult female rhesus macaque age was positively correlated with height (Spearman correlation, r=0.261, n=273, p<0.0005) (Figure S1). Additional calculations with different age cut-off points indicated that with a cut-off point of seven years the correlation was still significantly positive (Spearman correlation, r=0.162, n=210, p=0.019), but this disappeared when the sample encompassed only females above the age of eight (Spearman correlation, r=0.067, n=172, p=0.385). Linear regression showed that the relationship between age and height in adult female rhesus macaques was height (cm) = 51.461 + 0.068 * age (years). This means that adult rhesus females grow 0.068 cm per year and 0.68 cm in ten years, which we considered negligible relative to the range in height (about 45-58 cm) and ignored in the further analyses.

There was no significant correlation between age and height in adult female long-tailed macaques (Spearman correlation, r =-0.114, n=88, p=0.292).

Figure S1: *Height plotted against age for adult female rhesus macaques. The line shows a slight, but significant positive correlation between age and height. This relationship can be expressed as height (cm) = 51.461 + 0.068 * age (years).*

*Converting BMI in WHI-values*

According to Raman et al. 2005, BMI of solitary housed female macaques should be between 27 and 35 kg/m^2^. The average height of female rhesus macaques at the BPRC population is 52.19cm, while female long-tailed macaques are on average 44.31cm tall. See table for data conversion.

|  | BMI = kg/m^2^ | Average height | Average weight  (kg = BMI*m^2^) | WHI (kg/m^β^) |
| --- | --- | --- | --- | --- |
| Lower boundary rhesus ♀ | 27 | 52.19cm = 0.5219m | 27*0.5219^2^ = 27*0.272 = 7.35 kg | 7.35 / 0.5219^3^ = 51.73 = 52 kg/m^3^ |
| Upper boundary rhesus ♀ | 35 | 52.19cm = 0.5219m | 35*0.5219^2^ = 35*0.272 = 9.53 kg | 9.53 / 0.5219^3^ = 67.06 = 67 kg/m^3^ |
| Lower boundary long-tailed ♀ | 27 | 44.31cm = 0.4431m | 27*0.4431^2^ = 27*0.1963 = 5.30 kg | 5.30 / 0.4431^2.7^ = 47.73 = 48 kg/m^2.7^ |
| Upper boundary long-tailed ♀ | 35 | 44.31cm = 0.4431m | 35*0.4431^2^ = 35*0.1963 = 6.87 kg | 6.87 / 0.4431^2.7^ = 61.88 = 62 kg/m^2.7^ |

Table S1: *The age, body weight, height and WHI3.0 of four elderly female rhesus macaques*

| **Name** | **Age** | **Weight (kg)** | **Height (cm)** | **WHI3.0** |
| --- | --- | --- | --- | --- |
| Bertha | 34.5 | 5.1 | 46.2 | 51.72 |
| Wizco | 29.7 | 5.7 | 47.4 | 53.46 |
| Alarm | 26.9 | 6.9 | 47.8 | 63.18 |
| Mol | 25.7 | 8.1 | 49.3 | 67.60 |

Table S2: *Descriptive statistics of the studied populations (mean ± SEM)*

|  | **Current female** ♀  **rhesus macaques** | **Current male** ♂  **rhesus macaques** | **Current female** ♀  **long-tailed macaques** | **Current male** ♂  **long-tailed macaques** | **Founding female** ♀  **long-tailed macaques** | **Founding male** ♂  **long-tailed macaques** | **Wild female** ♀  **long-tailed macaques** | **Wild male** ♂  **long-tailed macaques** |
| --- | --- | --- | --- | --- | --- | --- | --- | --- |
| Sample size | N=273 | N=23 | N=92 | N=13 | N=24 | N=11 | N=9 | N=6 |
| Age (years) | 10.73 ± 0.26 | 13.21 ± 0.68 | 12.25 ± 4.35 | 12.07 ± 0.54 | 10.88 ± 0.86 | 10.40 ± 0.74 | 10.53 ± 1.17 | 11.09 ± 1.69 |
| Weight (kg) | 8.58 ± 0.10 | 12.03 ± 0.46 | 5.91 ± 1.19 | 9.86 ± 0.58 | 4.38 ± 0.22 | 6.65 ± 0.46 | 3.51 ± 0.13 | 5.29 ± 0.21 |
| Length (cm) | 52.19 ± 0.13 | 58.60 ± 0.43 | 44.31 ± 1.94 | 51.38 ± 0.64 | 41.22 ± 0.53 | 46.66 ± 1.06 | 42.11 ± 0.43 | 46.63 ± 0.43 |
| WHI^2.0^ (BMI) | 31.33 ± 0.30 | 35.04 ± 1.23 | 30.05 ± 4.69 | 37.24 ± 1.84 | 25.50 ± 0.89 | 30.28 ± 1.51 | 19.74 ± 0.39 | 24.29 ± 0.66 |
| WHI^2.7^ | NA | NA | 53.14 ± 8.14 | 59.41 ± 2.93 | 47.42 ± 1.57 | 51.69 ± 2.56 | 36.16 ± 0.60 | 41.43 ± 1.01 |
| WHI^3.0^ (Ponderal) | 60.05 ± 0.56 | 59.90 ± 2.20 | NA | NA | NA | NA | NA | NA |
| Abdominal circumference (cm) | 41.19 ± 0.40 | 45.50 ± 1.62 | 40.60 ± 5.57 | 44.59 ± 1.88 | 25.85 ± 0.68 | 26.33 ± 0.90 | 26.53 ± 1.03 | 26.71 ± 1.02 |
| Abdominal skinfold thickness (mm) | 6.46 ± 0.24 | 9.95 ± 1.62 | 6.55 ± 3.86 | 8.25 ± 1.76 | 2.54 ± 0.13 | 3.21 ± 0.51 | 1.58 ± 0.07 | 2.42 ± 0.21 |
| Subscapular skinfold thickness (mm) | 5.37 ± 0.12 | 4.67 ± 0.40 | 5.85 ± 1.81 | 7.23 ± 0.72 | 3.83 ± 0.17 | 4.25 ± 0.40 | 2.50 ± 0.06 | 3.52 ± 0.20 |
| Supra-iliac skinfold thickness (mm) | 6.10 ± 0.17 | 6.61 ± 0.83 | 7.86 ± 2.78 | 9.23 ± 1.10 | 4.09 ± 0.26 | 4.75 ± 0.66 | 2.37 ± 0.06 | 3.68 ± 0.21 |
| Triceps skinfold thickness (mm) | 2.25 ± 0.03 | 2.31 ± 0.09 | 2.12 ± 0.35 | 2.66 ± 0.20 | 1.16 ± 0.02 | 1.35 ± 0.11 | 1.06 ± 0.04 | 1.42 ± 0.06 |
| Total skinfold thickness (mm) | 20.15 ± 0.50 | 23.56 ± 2.79 | 22.27 ± 7.16 | 27.37 ± 3.57 | 10.46 ± 0.47 | 13.57 ± 1.59 | 7.50 ± 0.21 | 11.03 ± 0.63 |
| Body Condition Score | 3.19 ± 0.02 | 3.12 ± 0.12 | 3.15 ± 0.38 | 3.25 ± 0.12 | NA | NA | NA | NA |

Table S3: *Spearman correlations for body measurements indicating relative adiposity in adult male (n=23) and female (n=273) rhesus macaques that currently live in the BPRC breeding colony* ^†^. *Grey-marked areas represent non-significant results.*

| **Males**  **Females** | **Height** | **Weight** | **WHI^2.0^ (BMI)** | **WHI^3.0^ (Ponderal)** | **Abdominal circumference** | **Abdominal skinfold** | **Subscapular skinfold** | **Supra-iliac skinfold** | **Triceps skinfold** | **Total skinfold** | **Body Condition Score** |
| --- | --- | --- | --- | --- | --- | --- | --- | --- | --- | --- | --- |
| **Height** |  | r=0.245  n=23  p=0.260 | r=-0.171  n=23  p=0.435 | r=-0.380  n=23  p=0.074 | r=0.077  n=23  p=0.727 | r=-0.022  n=23  p=0.922 | r=-0.047  n=23  p=0.830 | r=0.124  n=23  p=0.574 | r=-0.275  n=23  p=0.204 | r=0.009  n=23  p=0.967 | r=-0.207  n=23  p=0.334 |
| **Weight** | r=0.634  n=273  p<0.0005 |  | r=0.858  n=23  p<0.0005 | r=0.739 n=23  p<0.0005 | r=0.833 n=23  p<0.0005 | r=0.659  n=23  p=0.001 | r=0.699 n=23  p<0.0005 | r=0.796 n=23  p<0.0005 | r=0.520  n=23  p=0.011 | r=0.775 n=23  p<0.0005 | r=0.514  n=23  p=0.012 |
| **WHI^2.0^ (BMI)** | r=0.276  n=273  p<0.0005 | r=0.902  n=273  p<0.0005 |  | r=0.956  n=23  p<0.0005 | r=0.842 n=23  p<0.0005 | r=0.771 n=23  p<0.0005 | r=0.791 n=23  p<0.0005 | r=0.792 n=23  p<0.0005 | r=0.686 n=23  p<0.0005 | r=0.845 n=23  p<0.0005 | r=0.695 n=23  p<0.0005 |
| **WHI^3.0^ (Ponderal)** | r=0.039  n=273  p=0.522 | r=0.766  n=273  p<0.0005 | r=0.965  n=273  p<0.0005 |  | r=0.745 n=23  p<0.0005 | r=0.699 n=23  p<0.0005 | r=0.737 n=23  p<0.0005 | r=0.724 n=23  p<0.0005 | r=0.652  n=23  p=0.001 | r=0.775 n=23  p<0.0005 | r=0.739 n=23  p<0.0005 |
| **Abdominal circumference** | r=0.416  n=269  p<0.0005 | r=0.885  n=269  p<0.0005 | r=0.890  n=269  p<0.0005 | r=0.812  n=269  p<0.0005 |  | r=0.887 n=23  p<0.0005 | r=0.743 n=23  p<0.0005 | r=0.777 n=23  p<0.0005 | r=0.600  n=23  p=0.002 | r=0.884 n=23  p<0.0005 | r=0.667  n=23  p=0.001 |
| **Abdominal skinfold** | r=0.184  n=271  p=0.002 | r=0.599  n=271 p<0.0005 | r=0.668  n=271  p<0.0005 | r=0.656  n=271  p<0.0005 | r=0.638  n=268  p<0.0005 |  | r=0.822 n=23  p<0.0005 | r=0.842 n=23  p<0.0005 | r=0.615  n=23  p=0.002 | r=0.958 n=23  p<0.0005 | r=0.723 n=23  p<0.0005 |
| **Subscapular skinfold** | r=0.224  n=271  p<0.0005 | r=0.662  n=271 p<0.0005 | r=0.730  n=271  p<0.0005 | r=0.711  n=271  p<0.0005 | r=0.669  n=268  p<0.0005 | r=0.714  n=271  p<0.0005 |  | r=0.833 n=23  p<0.0005 | r=0.687 n=23  p<0.0005 | r=0.890 n=23  p<0.0005 | r=0.734 n=23  p<0.0005 |
| **Supra-iliac skinfold** | r=0.180  n=271  p=0.003 | r=0.627  n=271 p<0.0005 | r=0.697  n=271  p<0.0005 | r=0.683  n=271  p<0.0005 | r=0.638  n=268  p<0.0005 | r=0.764  n=271  p<0.0005 | r=0.817  n=271  p<0.0005 |  | r=0.700 n=23  p<0.0005 | r=0.944 n=23  p<0.0005 | r=0.708 n=23  p<0.0005 |
| **Triceps skinfold** | r=0.222  n=270  p<0.0005 | r=0.560  n=270  p<0.0005 | r=0.588  n=270  p<0.0005 | r=0.556  n=270  p<0.0005 | r=0.551  n=267  p<0.0005 | r=0.544  n=270  p<0.0005 | r=0.591  n=270  p<0.0005 | r=0.549  n=270  p<0.0005 |  | r=0.696  n=23  p<0.0005 | r=0.498  n=23  p=0.016 |
| **Total skinfold** | r=0.205  n=270  n=0.001 | r=0.675  n=270  p<0.0005 | r=0.749  n=270  p<0.0005 | r=0.732  n=270  p<0.0005 | r=0.693  n=267  p<0.0005 | r=0.929  n=270  p<0.0005 | r=0.875  n=270  p<0.0005 | r=0.914  n=270  p<0.0005 | r=0.637  n=270  p<0.0005 |  | r=0.767  n=23  p<0.0005 |
| **Body Condition Score** | r=0.317  p=273  p<0.0005 | r=0.710  n=274  p<0.0005 | r=0.716  n=273  p<0.0005 | r=0.659  n=273  p<0.0005 | r=0.669  n=269  p<0.0005 | r=0.609  n=271  p<0.0005 | r=0.541  n=271  p<0.0005 | r=0.635  n=271  p<0.0005 | r=0.460  n=270  p<0.0005 | r=0.657  n=270  p<0.0005 |  |

^†^ Correlations for males appear in the upper-right triangle and those for females appear in the lower-left triangle. Numbers represent the Spearman correlation coefficient (r), sample size (n) and level of significance (p-value).

Table S4: *Spearman correlations for body measurements indicating relative adiposity in adult male (n=13) and female (n=92) long-tailed macaques that currently live in the BPRC breeding colony* ^†^. *Grey-marked areas represent non-significant results.*

| **Males**  **Females** | **Height** | **Weight** | **WHI^2.0^ (BMI)** | **WHI^2.7^** | **Abdominal circumference** | **Abdominal skinfold** | **Subscapular skinfold** | **Supra-iliac skinfold** | **Triceps skinfold** | **Total skinfolds** | **Body Condition Score** |
| --- | --- | --- | --- | --- | --- | --- | --- | --- | --- | --- | --- |
| **Height** |  | r=0.327  n=13  p=0.275 | r=-0.063  n=13  p=0.837 | r=-0.157  n=13  p=0.609 | r=0.448  n=12  p=0.144 | r=-0.056  n=12  p=0.863 | r=0.186  n=12  p=0.564 | r=0.172  n=12  p=0.594 | r=0.224  n=12  p=0.484 | r=-0.053  n=12  p=0.871 | r=-0.060  n=13  p=0.846 |
| **Weight** | r=0.607  n=88  p<0.0005 |  | r=0.879  n=13  p<0.0005 | r=0.835  n=13  p<0.0005 | r=0.895 n=12  p<0.0005 | r=0.657  n=12  p=0.020 | r=0.818  n=12  p=0.001 | r=0.881  n=12  p<0.0005 | r=0.727  n=12  p=0.007 | r=0.734  n=12  p=0.007 | r=0.830  n=13  p<0.0005 |
| **WHI^2.0^ (BMI)** | r=0.223  n=88  p=0.037 | r=0.887  n=88  p<0.0005 |  | r=0.978  n=13  p<0.0005 | r=0.811  n=12  p=0.001 | r=0.734  n=12  p=0.007 | r=0.860  n=12  p<0.0005 | r=0.895  n=12  p<0.0005 | r=0.727  n=12  p=0.007 | r=0.839  n=12  p=0.001 | r=0.898  n=13  p<0.0005 |
| **WHI^2.7^** | r=-0.041  n=88  p=0.706 | r=0.782  n=88  p<0.0005 | r=0.976  n=88  p<0.0005 |  | r=0.769  n=12  p=0.003 | r=0.804  n=12  p=0.002 | r=0.846  n=12  p=0.001 | r=0.923  n=12  p<0.0005 | r=0.685  n=12  p=0.014 | r=0.881  n=12  p<0.0005 | r=0.944  n=13  p<0.0005 |
| **Abdominal circumference** | r=0.363  n=84  p=0.001 | r=0.835  n=87  p<0.0005 | r=0.846  n=84  p<0.0005 | r=0.808  n=84  p<0.0005 |  | r=0.741  n=12  p=0.006 | r=0.881  n=12  p<0.0005 | r=0.888  n=12  p<0.0005 | r=0.790  n=12  p=0.002 | r=0.811  n=12  p=0.001 | r=0.785  n=12  p=0.002 |
| **Abdominal skinfold** | r=0.383  n=85  p<0.0005 | r=0.546  n=87  p<0.0005 | r=0.511  n=85  p<0.0005 | r=0.464 n=85  p<0.0005 | r=0.614 n=84  p<0.0005 |  | r=0.874 n=12  p<0.0005 | r=0.839 n=12  p=0.001 | r=0.818  n=12  p=0.001 | r=0.958 n=12  p<0.0005 | r=0.864 n=12  p<0.0005 |
| **Subscapular skinfold** | r=0.201  n=86  p=0.064 | r=0.550  n=88  p<0.0005 | r=0.574  n=86  p<0.0005 | r=0.540 n=86  p<0.0005 | r=0.633  n=85  p<0.0005 | r=0.552  n=87  p<0.0005 |  | r=0.916 n=12  p<0.0005 | r=0.867 n=12  p<0.0005 | r=0.944 n=12  p<0.0005 | r=0.853 n=12  p<0.0005 |
| **Supra-iliac skinfold** | r=0.347  n=85  p=0.001 | r=0.572  n=87  p<0.0005 | r=0.517  n=85  p<0.0005 | r=0.457 n=85  p<0.0005 | r=0.641 n=84  p<0.0005 | r=0.465  n=87  p<0.0005 | r=0.711  n=87  p<0.0005 |  | r=0.762  n=12  p=0.004 | r=0.923  n=12  p<0.0005 | r=0.961  n=12  p<0.0005 |
| **Triceps skinfold** | r=0.175  n=86  p=0.108 | r=0.493  n=88  p<0.0005 | r=0.557  n=86  p<0.0005 | r=0.519  n=86  p<0.0005 | r=0.600 n=85  p<0.0005 | r=0.410  n=87  p<0.0005 | r=0.632  n=88  p<0.0005 | r=0.602  n=87  p<0.0005 |  | r=0.825  n=12  p=0.001 | r=0.728  n=12  p=0.007 |
| **Total skinfolds** | r=0.359  n=85  p=0.001 | r=0.650  n=87  p<0.0005 | r=0.624  n=85  p<0.0005 | r=0.570 n=85  p<0.0005 | r=0.734 n=84  p<0.0005 | r=0.824  n=87  p<0.0005 | r=0.847  n=87  p<0.0005 | r=0.822 n=87  p<0.0005 | r=0.624 n=87  p<0.0005 |  | r=0.914  n=12  p<0.0005 |
| **Body Condition Score** | r=0.226  n=88  p=0.034 | r=0.595  n=92  p<0.0005 | r=0.585  n=88  p<0.0005 | r=0.544  n=88  p<0.0005 | r=0.539  n=87  p<0.0005 | r=0.473  n=87  p<0.0005 | r=0.488  n=88  p<0.0005 | r=0.459 n=87  p<0.0005 | r=0.414 n=88  p<0.0005 | r=0.558 n=87  p<0.0005 | X |

^†^ Correlations for males appear in the upper-right triangle and those for females appear in the lower-left triangle. Numbers represent the Spearman correlation coefficient (r), sample size (n) and level of significance (p-value).

Table S5: *Spearman correlations for body measurements indicating relative adiposity in adult male (n=11) and female (n=24) long-tailed macaques that founded the current BPRC breeding population* ^†^. *Grey-marked areas represent non-significant results.*

| **Males**  **Females** | **Height** | **Weight** | **WHI^2.0^ (BMI)** | **WHI^2.7^** | **Abdominal circumference** | **Abdominal skinfold** | **Subscapular skinfold** | **Supra-iliac skinfold** | **Triceps skinfold** | **Total skinfolds** |
| --- | --- | --- | --- | --- | --- | --- | --- | --- | --- | --- |
| **Height** |  | r=0.728  n=11  p=0.011 | r=0.323  n=11  p=0.332 | r=-0.023  n=11  p=0.947 | r=0.556  n=11  p=0.076 | r=-0.354  n=11  p=0.286 | r=0.173  n=11  p=0.611 | r=0.105  n=11  p=0.759 | r=0.077  n=11  p=0.821 | r=-0.073  n=11  p=0.831 |
| **Weight** | r=0.747  n=24  p<0.0005 |  | r=0.829  n=11  p=0.002 | r=0.533  n=11  p=0.091 | r=0.911  n=11  p<0.0005 | r=0.032  n=11  p=0.926 | r=0.560  n=11  p=0.073 | r=0.621  n=11  p=0.041 | r=0.601  n=11  p=0.050 | r=0.432  n=11  p=0.185 |
| **WHI^2.0^ (BMI)** | r=0.297  n=24  p=0.158 | r=0.837  n=24  p<0.0005 |  | r=0.882  n=11  p<0.0005 | r=0.918  n=11  p<0.0005 | r=0.337  n=11  p=0.311 | r=0.755  n=11  p=0.007 | r=0.902  n=11  p<0.0005 | r=0.836  n=11  p=0.001 | r=0.729  n=11  p=0.011 |
| **WHI^2.7^** | r=0.051  n=24  p=0.813 | r=0.661  n=24  p<0.0005 | r=0.952  n=24  p<0.0005 |  | r=0.718  n=11  p=0.013 | r=0.638  n=11  p=0.035 | r=0.827  n=11  p=0.002 | r=0.907  n=11  p<0.0005 | r=0.909  n=11  p<0.0005 | r=0.861  n=11  p=0.001 |
| **Abdominal circumference** | r=0.323  n=24  p=0.124 | r=0.757  n=24  p<0.0005 | r=0.829  n=24  p<0.0005 | r=0.763 n=24  p<0.0005 |  | r=0.223  n=11  p=0.509 | r=0.682  n=11  p=0.021 | r=0.806  n=11  p=0.003 | r=0.691  n=11  p=0.019 | r=0.588  n=11  p=0.057 |
| **Abdominal skinfold** | r=0.096  n=24  p=0.656 | r=0.542  n=24  p=0.006 | r=0.743  n=24  p<0.0005 | r=0.769  n=24  p<0.0005 | r=0.441  n=24  p=0.031 |  | r=0.743  n=11  p=0.009 | r=0.614  n=11  p=0.044 | r=0.415  n=11  p=0.205 | r=0.849  n=11  p=0.001 |
| **Subscapular skinfold** | r=0.335  n=24  p=0.109 | r=0.736  n=24  p<0.0005 | r=0.818  n=24  p<0.0005 | r=0.749  n=24  p<0.0005 | r=0.726  n=24  p<0.0005 | r=0.484  n=24  p=0.017 |  | r=0.834  n=11  p=0.001 | r=0.691  n=11  p=0.019 | r=0.879  n=11  p<0.0005 |
| **Supra-iliac skinfold** | r=0.142  n=24  p=0.507 | r=0.631  n=24  p=0.001 | r=0.822  n=24  p<0.0005 | r=0.838  n=24  p<0.0005 | r=0.788  n=24  p<0.0005 | r=0.548  n=24  p=0.006 | r=0.823  n=24  p<0.0005 |  | r=0.720  n=11  p=0.013 | r=0.900  n=11  p<0.0005 |
| **Triceps skinfold** | r=0.078  n=24  p=0.717 | r=0.355  n=24  p=0.089 | r=0.408  n=24  p=0.048 | r=0.413  n=24  p=0.045 | r=0.133  n=24  p=0.535 | r=0.454  n=24  p=0.026 | r=0.239  n=24  p=0.261 | r=0.270  n=24  p=0.201 |  | r=0.651  n=11  p=0.030 |
| **Total skinfolds** | r=0.234  n=24  p=0.270 | r=0.724  n=24  p<0.0005 | r=0.884  n=24  p<0.0005 | r=0.871  n=24  p<0.0005 | r=0.766  n=24  p<0.0005 | r=0639  n=24  p=0.001 | r=0.939  n=24  p<0.0005 | r=0.930  n=24  p<0.0005 | r=0.300  n=24  p=0.155 |  |

^†^ Correlations for males appear in the upper-right triangle and those for females appear in the lower-left triangle. Numbers represent the Spearman correlation coefficient (r), sample size (n) and level of significance (p-value).

Table S6: *Spearman correlations for body measurements indicating relative adiposity in wild adult male (n=6) and female (n=9) long-tailed macaques ^†^. Grey-marked areas represent non-significant results. Grey-marked areas represent non-significant results.*

| **Males**  **Females** | **Height** | **Weight** | **WHI^2.0^ (BMI)** | **WHI^2.7^** | **Abdominal circumference** | **Abdominal skinfold** | **Subscapular skinfold** | **Supra-iliac skinfold** | **Triceps skinfold** | **Total skinfolds** |
| --- | --- | --- | --- | --- | --- | --- | --- | --- | --- | --- |
| **Height** |  | r=0.829  n=6  p=0.042 | r=0.429  n=6  p=0.397 | r=0.143  n=6  p=0.787 | r=0.257  n=6  p=0.623 | r=0.290  n=6  p=0.577 | r=0.696  n=6  p=0.125 | r=0.464  n=6  p=0.354 | r=-0.319  n=6  p=0.538 | r=0.314  n=6  p=0.544 |
| **Weight** | r=0.837  n=9  p=0.005 |  | r=0.829  n=6  p=0.042 | r=0.657  n=6  p=0.156 | r=0.314  n=6  p=0.544 | r=0.667  n=6  p=0.148 | r=0.928  n=6  p=0.008 | r=0.812  n=6  p=0.050 | r=-0.116  r=6  p=0.827 | r=0.714  n=6  p=0.111 |
| **WHI^2.0^ (BMI)** | r=0.569  n=9  p=0.110 | r=0.900  n=9  p=0.001 |  | r=0.943  n=6  p=0.005 | r=0.543  n=6  p=0.266 | r=0.754  n=6  p=0.084 | r=0.783  n=6  p=0.066 | r=0.812  n=6  p=0.050 | r=-0.029  n=6  p=0.957 | r=0.771  n=6  p=0.072 |
| **WHI^2.7^** | r=0.226  n=9  p=0.559 | r=0.683  n=9  p=0.042 | r=0.917  n=9  p=0.001 |  | r=0.429  n=6  p=0.397 | r=0.812  n=6  p=0.050 | r=0.696  n=6  p=0.125 | r=0.812  n=6  p=0.050 | r=0.203  n=6  p=0.700 | r=0.829  n=6  p=0.042 |
| **Abdominal circumference** | r=0.414  n=9  p=0.269 | r=0.714  n=9  p=0.031 | r=0.672  n=9  p=0.047 | r=0.672  n=9  p=0.047 |  | r=0.319  n=6  p=0.538 | r=0.232  n=6  p=0.658 | r=0.261  n=6  p=0.618 | r=-0.116  n=6  p=0.827 | r=0.200  n=6  p=0.704 |
| **Abdominal skinfold** | r=0.697  n=9  p=0.037 | r=0.485  n=9  p=0.186 | r=0.323  n=9  p=0.396 | r=0.034  n=9  p=0.931 | r=-0.056  n=9  p=0.887 |  | r=0.868  n=6  p=0.025 | r=0.971  n=6  p=0.001 | r=0.603  n=6  p=0.205 | r=0.986  n=6  p<0.0005 |
| **Subscapular skinfold** | r=0.722  n=9  p=0.028 | r=0.462  n=9  p=0.210 | r=0.269  n=9  p=0.484 | r=-0.017  n=9  p=0.966 | r=-0.042  n=9  p=0.914 | r=0.837  n=9  p=0.005 |  | r=0.956  n=6  p=0.003 | r=0.250  n=6  p=0.633 | r=0.899  n=6  p=0.015 |
| **Supra-iliac skinfold** | r=0.672  n=9  p=0.047 | r=0.816  n=9  p=0.007 | r=0.869  n=9  p=0.002 | r=0.727  n=9  p=0.026 | r=0.376  n=9  p=0.319 | r=0.557  n=9  p=0.119 | r=0.452  n=9  p=0.222 |  | r=0.441  n=6  p=0.381 | r=0.986  n=6  p<0.0005 |
| **Triceps skinfold** | r=0.518  n=9  p=0.153 | r=0.507  n=9  p=0.153 | r=0.324  n=9  p=0.396 | r=0.175  n=9  p=0.653 | r=0.384  n=9  p=0.308 | r=0.692  n=9  p=0.039 | r=0.556  n=9  p=0.120 | r=0.307  n=9  p=0.422 |  | r=0.551  n=6  p=0.257 |
| **Total skinfolds** | r=0.731  n=9  p=0.025 | r=0.577  n=9  p=0.104 | r=0.410  n=9  p=0.273 | r=0.142  n=9  p=0.715 | r=0.114  n=9  p=0.770 | r=0.975  n=9  p<0.0005 | r=0.861  n=9  p=0.003 | r=0.579  n=9  p=0.103 | r=0.808  n=9  p=0.008 |  |

^†^ Correlations for males appear in the upper-right triangle and those for females appear in the lower-left triangle. Numbers represent the Spearman correlation coefficient (r), sample size (n) and level of significance (p-value).

Table S7*: Results of the statistical tests in which the effect of sex (male or female) and origin of the population (wild, founder or current) on different long-tailed macaque body measures were tested. Population differences were tested with one-way ANOVAs or Kruskal-Wallis H-tests, for males and females separately. Sex differences were tested with independent samples t-tests or Mann-Whitney U-test, for the different populations separately. Grey-marked areas represent non-significant results.*

|  | **Rhesus macaques** | **Long-tailed macaques** | | | | |
| --- | --- | --- | --- | --- | --- | --- |
| **Measure** | **Sex ^†^ – current population (n=296)** | **Population origin** ^‡^ **– data of females (n=125)** | **Population origin** ^‡^ **– data of males (n=30)** | **Sex ^†^ – current population (n=105)** | **Sex ^†^ – founder population (n=35)** | **Sex ^†^ – wild population (n=15)** |
| **Body weight** | U=485.5, n=296, p<0.0005  M > F | H(2)=41.891, p<0.0005  Cu > Fo ~ Wi  (p<0.0005; p<0.0005; p=0.306) | H(2)=18.418, p<0.0005  Cu > Fo ~ Wi  (p=0.009; p<0.0005; p=0.396) | t=6.712, n=105, p<0.0005  M > F | t=5.088, n=35, p<0.0005  M > F | t=7.779, n=15, p<0.0005  M > F |
| **Height** | t=13.884, n=296, p<0.0005  M > F | F(2,120)=23.790, p<0.0005  Cu > Fo ~ Wi  (p<0.0005; p=0.008; p=0.798) | F(2,120)=13.541, p=0.001  Cu > Fo ~ Wi  (p=0.005; p=0.009; p=1.000) | t=11.960, n=101, p<0.0005  M > F | t=5.142, n=35, p<0.0005  M > F | t=7.088, n=15, p<0.0005  M > F |
| **BMI** | U=2022, n=296, p=0.005  M > F | H(2)=35.302, p<0.0005  Cu > Fo > Wi  (p<0.0005; p=0.001; p=0.042) | H(2)=15.952, p<0.0005  Cu ~ Fo; Cu > Wi; Fo ~ Wi  (p=0.065; p<0.0005; p=0.160) | t=3.767, n=101, p=0.002  M > F | U=59, n=35, p=0.008  M > F | t=6.376, n=15, p<0.0005  M > F |
| **WHI** | U=2958, n=296, p=0.645  M ~ F | H(2)=29.869, p<0.0005  Cu > Fo > Wi  (p=0.017; p<0.0005; p=0.011) | H(2)=12.284, p=0.002  Cu ~ Fo; Cu > Wi; Fo ~ Wi  (p=0.393; p=0.001; p=0.087) | t=2.489, n=101, p=0.014  M > F | U=95, n=35, p=0.198  M ~ F | t=4.776, n=15, p<0.0005  M > F |
| **Abdominal circumference** | U=2171, n=292, p=0.018 | H(2)=67.688, p<0.0005  Cu > Fo ~ Wi  (p<0.0005; p<0.0005; p=1.000) | H(2)=20.442, p<0.0005  Cu > Fo ~ Wi  (p=0.003; p<0.0005; p=1.000) | U=375.5, n=99, p=0.116  M ~ F | t=0.409, n=35, p=0.685  M ~ F | t=0.119, n=15, p=0.907  M ~ F |
| **Abdominal skinfold thickness** | U=2411.5, n=292, p=0.079  M ~ F | H(2)=58.631, p<0.0005  Cu > Fo ~ Wi  (p<0.0005; p<0.0005; p=0.272) | H(2)=7.951, p=0.019  Cu ~ Fo; Cu > Wi; Fo ~ Wi  (p=0.108; p=0.031; p=1.000) | U=482, n=99, p=0.668  M ~ F | U=97, n=35, p=0.224  M ~ F | t=4.371, n=15, p=0.001  M > F |
| **Subscapular skinfold thickness** | U=2123, n=293, p=0.012  M < F | H(2)=47.212, p<0.0005  Cu > Fo ~ Wi  (p<0.0005; p<0.0005; p=0.112) | H(2)=16.204, p<0.0005  Cu > Fo ~ Wi  (p=0.014; p = 0.001; p=0.520) | U=367.5, n=100, p=0.089  M ~ F | U=101, n=35, p=0.283  M ~ F | t=4.787, n=15, p=0.003  M > F |
| **Supra-iliac skinfold thickness** | U=3088.5, n=293, p=0.966  M ~ F | H(2)=56.373, p<0.0005  Cu > Fo ~ Wi  (p<0.0005; p<0.0005; p=0.209) | H(2)=14.722, p=0.001  Cu > Fo ~ Wi  (p=0.006; p=0.003; p=1.000) | U=416, n=99, p=0.256  M ~ F | U=107.5, n=35, p=0.390  M ~ F | U=0, n=15, p<0.0005  M > F |
| **Triceps skinfold thickness** | U=2804.5, n=292, p=0.457  M ~ F | H(2)=71.613, p<0.0005  Cu > Fo ~ Wi  (p<0.0005; p<0.0005; p=1.000) | H(2)=18.233, p<0.0005  Cu > Fo ~ Wi  (p=0.009; p<0.0005; p=1.000) | U=250.5, n=100, p=0.003  M > F | t=1.716, n=35, p=0.115  M ~ F | t=4.935, n=15, p<0.0005  M > F |
| **Total skinfold thickness** | U=2901.5, n=292, p=0.621  M ~ F | H(2)=66.320, p<0.0005  Cu > Fo ~ Wi  (p<0.0005; p<0.0005; p=0.535) | H(2)=16.039, p<0.0005  Cu > Fo ~ Wi  (p=0.005; p=0.001; p=1.000) | U=411, n=99, p=0.234  M ~ F | U=71.5, n=35, p=0.030  M > F | t=6.214, n=15, p<0.0005  M > F |
| **Body Condition Score** | U=2598, n=296, p=0.148  M ~ F |  |  | U=535.5, n=105, p=0.523  M ~ F |  |  |

^†^ M = male, F = female

^‡^ Cu = current population, Fo = founder population, Wi = Wild population, the p-values between brackets show outcomes of the post-hoc comparisons with Bonferroni correction of Cu vs. Fo, Cu vs. Wi and Fo vs. Wi, respectively.

Figure S2: *BMI plotted against height for adult rhesus macaques. Solid dots represent females, while solid triangles represent males. Height was positively correlated with BMI in female rhesus macaques (black solid line), but not in males (dashed black line).*

Figure S3: *BMI plotted against height for adult long-tailed macaques currently living at the BPRC breeding colony. Solid dots represent females, while solid triangles represent males.*
